# Complete mitochondrial genome of *Proisotoma minuta* (Collembola: Proisotominae)

**DOI:** 10.1080/23802359.2022.2068977

**Published:** 2022-04-29

**Authors:** Yue Tian, Xiulian Miao, Shilin Song, Zhengmin Zhang, Shiran Hu, Deli Wei, Meng Wang

**Affiliations:** a School of Life Sciences, Liaocheng University, Liaocheng, China; b School of Ji Xianlin Honors, Liaocheng University, Liaocheng, China; c Department of Reproductive Medicine, Liaocheng Peoples’ Hospital, Liaocheng, China

**Keywords:** Proisotominae, mitochondrial genome, *Proisotoma minuta*, phylogeny

## Abstract

The springtail *Proisotoma minuta* is a cosmopolitan species that can be found in many different habitats, especially in soil ecosystems. It is considered to be a good indicator of soil health. In this study, mitogenome information was obtained, which could lay a foundation for future fauna studies. The mitogenome of *P. minuta* is a circular module of 15,930 bp, including 13 protein-coding genes, 22 transfer RNA genes, and 2 ribosomal RNA genes. The mitogenome of *P. minuta* is composed of 35.9% A, 28.5% T, 13.7% G, and 21.3% C. Phylogenetic analysis revealed that *P. minuta* was well grouped in the subfamily Proisotominae and had a closer relationship with Anurophorinae than Isotominae subfamily and other families.

The springtail *Proisotoma minuta* (Tullberg, 1871) can be found in various ecological environments and it causes damage to plant crown (Nematollahi et al. 2009). It has been considered an important candidate bioindicator to assess soil quality spiked with trace metals. *Proisotoma minuta* shows high sensitivity to cadmium, copper, and zinc, but it could tolerate high levels of lead (Nursita et al. 2005), arsenic (Greenslade and Vaughan 2003), and mercury (Buch et al. 2016). *Proisotoma minuta* may play a major role in the biological control of plant pathogens, such as *Rhizoctonia solani*, due to its mycophagous characteristic (Lartey et al. 1991). Here, the mitochondrial genome of *P. minuta* was assembled, which could be helpful for assessing its systematic position.

A voucher specimen of *P. minuta* was collected from forest soil with a humus layer of deciduous leaves at Wulian Mountain, Rizhao City, China (35.687°N, 119.395°E). The species is abundant in the field soil and does not belong to the list of key protected wild animals in China. Permission to enter Wulian Mountain for a field survey in July 2019 was acquired from the park service. After morphological identification, the collected collembolans were cultured in a laboratory environment following the standardized conditions described previously (Buch et al. 2016) and then stored in the laboratory of Animal Museum, School of Life Sciences, Liaocheng University (http://smkxxy.lcu.edu.cn, contact person: Xiulian Miao; email: miaoxiulian@lcu.edu.cn), under voucher no. TC202008. Total genomic DNA was extracted from the collected springtail individuals by using the CTAB method and then fragmented. The sequencing library was prepared using the Nebnext Ultra DNA Library Prep Kit for Illumina and sequenced with Illumina NovaSeq. Approximately 4.2 Gbp of clean data was obtained in this study. The mitogenome was assembled with SPAdes version 3.13.0 (Nurk et al. 2013) with the parameter ‘-k 127’, annotated with MITOS2 (Bernt et al. 2013) and deposited in GenBank with the accession number MW874475.

The complete mitogenome of *P. minuta* (Genbank accession no. MW874475) was sequenced to be 15,930 bp in size. The mitogenome consisted of 13 typical protein-coding genes (PCGs), 22 transfer RNA genes (tRNAs), and 2 ribosomal RNA genes (rRNAs), similar to the typical mitogenome of other collembolan arthropods, such as *Folsomia candida* (KU198392) and *Isotomurus maculatus* (MK509021), but it has a different gene order compared with *Pseudachorutes palmiensis* (Dong et al. 2020). In *P. minuta* mitogenome, 23 genes were encoded on the H-strand, while the other 14 genes were encoded on the L-strand. The mean length of tRNAs was 64 bp, ranging from 60 to 71 bp. The overall base composition was 35.9% of A, 28.5% of T, 13.7% of G, and 21.3% of C, demonstrating a bias of higher AT content (64.4%). All PCGs started with a typical ATN codon, including seven with ATG, three with ATA, and three with ATT. Nine PCGs used TAA as the stop codon, two PCGs (*atp8* and *nad1*) used TAG, and two (*cox2* and *cox3*) used incomplete T.

For validation of the phylogenetic position of *P. minuta* in Collembola, a maximum-likelihood (ML) tree was constructed on the basis of 20 Collembola mitogenome sequences, including *P. minuta* and one Diplura specie mitogenome sequence as an outgroup, by using MEGA 7.0 (Kumar et al. 2016) with the Kimura 2-parameter model and gamma-distributed with invariant sites (G + I). The robustness was tested with 2000 bootstraps. The ML tree showed that *P. minuta* was clustered within the subfamily Proisotominae, which is more closely related to the subfamily Anurophorinae than Isotominae subfamily and other families (Figure 1).

**Figure 1.**
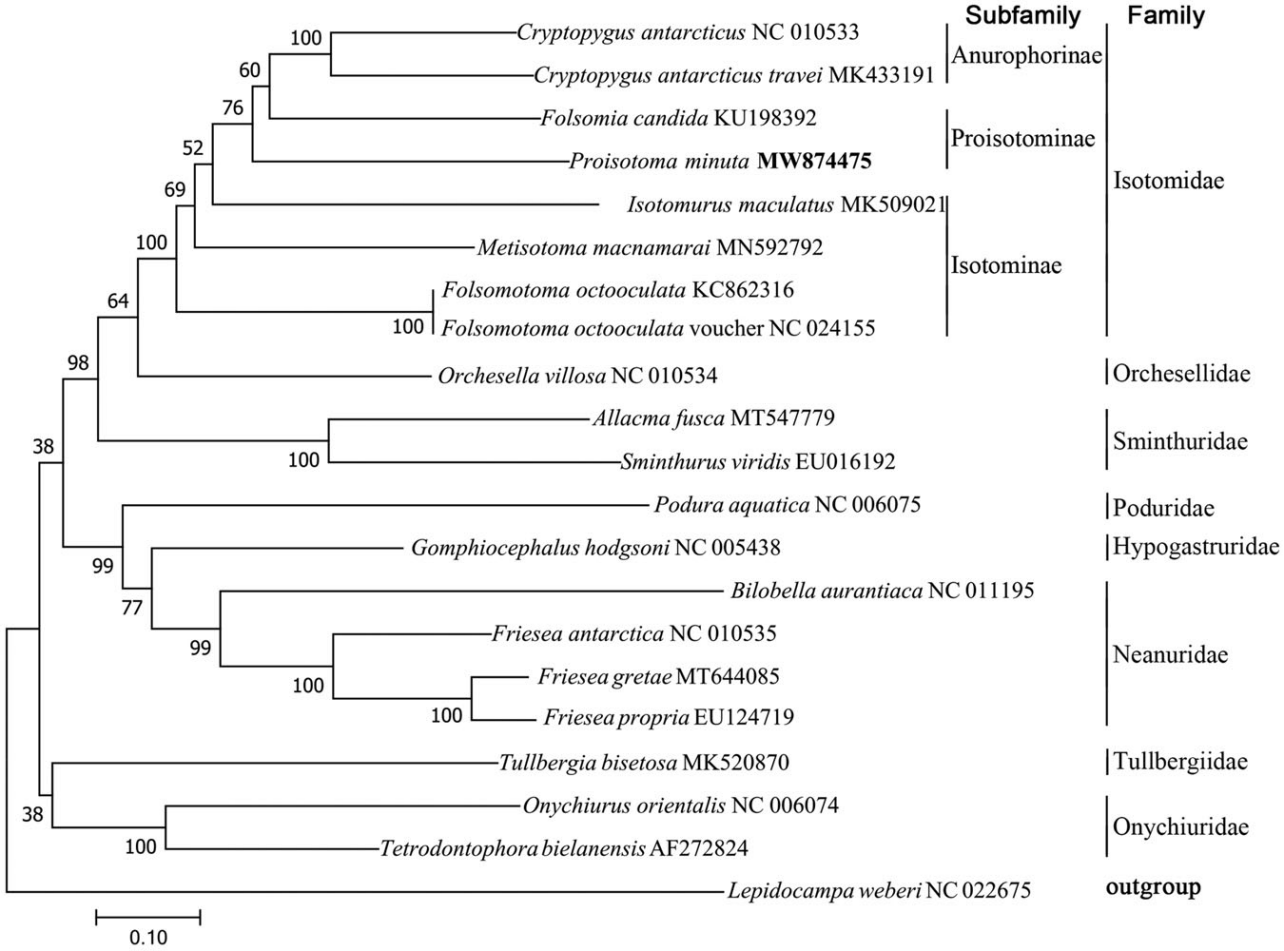
Phylogenetic tree obtained from 21 mitogenome sequences. The tree was generated using maximum-likelihood analysis with Kimura 2-parameter model and gamma distributed with invariant sites (G + I). The robustness of the tree was tested with 2000 bootstraps. The numbers on the branches indicate bootstrap values.

## Data Availability

The data that support the findings of this study are openly available in the GenBank of NCBI at https://www.ncbi.nlm.nih.gov under the accession no. MW874475. The associated BioProject, SRA and Bio-sample numbers are PRJNA770163, SRR16296415, and SAMN22210612, respectively.
